# Endothelial Injury in Diving: Atomic Force-, Electronic-, and Light-Microscopy Study of the Ovine Decompressed Blood Vessels

**DOI:** 10.3389/fphys.2021.767435

**Published:** 2021-10-15

**Authors:** Ran Arieli

**Affiliations:** ^1^Israel Naval Medical Institute, Israel Defense Forces Medical Corps, Haifa, Israel; ^2^Eliachar Research Laboratory, Western Galilee Medical Center, Nahariya, Israel

**Keywords:** active hydrophobic spot AHS, bubbles, detachment, nanobubble, endothelium

## Abstract

We suggested that the nanobubbles, which appear at the active hydrophobic spots (AHSs) at the luminal aspect of the blood vessels, are the gas micronuclei from which the decompression bubbles evolve and the endothelial injury during the decompression is due to the tearing off the cell membranes with the detaching bubbles. Ovine blood vessels were stretched over the polycarbonate plates or glass microscopic slides and were exposed under saline to the hyperbaric pressure (1,013 kPa, 19 h). Following decompression, the blood vessels were photographed for the identification (by bubble formation) of the AHS. Nanobubbles could not be demonstrated at the AHS by using the atomic force microscopy (AFM) because of the roughness of the surface, which disabled the close contact of the probe. In the electron microscopy, no endothelial cells were observed in the samples from the area near to the AHS, but the underlying elastin layer of the intima was observed adjacent to the media. Some intact endothelial cells were observed only in the locations far from an AHS. In the optical microscopy, no endothelial cells were observed in the blood vessels in close proximity to the AHS and in some sections, debris or a detached cluster of the endothelial cells were observed. Intact endothelial cells could be found at the sites distant from an AHS. This study supports the assumption, where the detached bubbles tear off the endothelial cells and cause the initial endothelial injury following the decompression.

## Introduction

In the search for the hypothesized gas micronuclei from which the bubbles evolve during the decompression after diving, we succeeded in establishing the chain of the events (Arieli, 2017). The lung surfactant such as dipalmitoylphosphatidylcholine (DPPC) leaks into the bloodstream. By leaving the plasma, the DPPC settles on the luminal aspect of the blood vessels to create an oligolamellar lining of the phospholipids (Hills, 1992). We named this site an “active hydrophobic spot” (AHS). The nanobubbles formed at the AHS, regardless of the diving activity, from the dissolved gas, become the gas micronuclei from which the bubbles evolve on the decompression. Decompression bubbles detach from the AHS at a force of 4.5 × 10^–6^ N, which is lower than the adhesion hydrophobic force of 38 × 10^–6^ N. In addition to the other considerations (Arieli and Marmur, 2017), led us to suggest that a bubble detaches with a piece of the underlying membrane. The adhesion force between the membrane bilayer is low and may cavitate by exposure to the clinical ultrasound (Krasovitski et al., 2011). Thus, this study related the endothelial injury during the decompression to the tearing off of the cell membranes with the detaching bubbles. This mechanism is not surprising, since it is well accepted to flush bubbles for descementing the endothelial cells for the preparation of the cell culture or implantation (Ruzza et al., 2015). Endothelial injury during the decompression is well established and was even suggested as the main cause of the decompression illness (DCI) (Madden and Laden, 2009; Madden et al., 2010; Lambrechts et al., 2013; Zhang et al., 2016, 2017a,b). The aim of this study was to delve into the microworld of the bubbles and the endothelial cells in the blood vessels of the sheep.

## Research Design and Methods

Initially, an effort was made to watch the nanobubbles, which were supposed to form at the AHS with the aid of the atomic force microscopy (AFM). Nanobubbles were detected at any tested artificial hydrophobic surface underwater, but not yet at the hydrophobic animated tissue. The second phase was to observe the damage to the endothelial cells by detaching the bubbles by using the electron microscopy (EM). This study expected to observe the breaches in the AHS oligolamellar layer. In the third phase, this study observed the sections from the blood vessels at the AHS and controls the samples by using light microscopy (LM). Each phase of the study was dictated by the previous one.

### Tissue Preparation Common to the Three Phases

The methods were described in detail in the previous studies (Arieli and Marmur, 2016; Arieli et al., 2016, 2019) and will be presented briefly here.

The complete heart and lungs from 10 slaughtered sheep (harvested on separate days) were obtained at the abattoir. In the laboratory, under the saline and without any exposure to air, this study sampled the four blood vessels such as the aorta, superior vena cava, pulmonary vein, and pulmonary artery. For the AFM study, the blood vessels were clamped on both sides denying contact to air from the interior. They were then extracted from the saline and the external surface was wiped dry with the filter paper. Two polycarbonate plates of 2.5 cm × 2.5 cm were glued together by using cyanoacrylate adhesive. Then, under saline, the two plates were cut free. For the electron and light microscopy, the blood vessels were gently stretched on the microscope slides held by the metal clips with the luminal aspect exposed. Eight polycarbonate plates or four slides were placed anaerobically at the bottom of a rectangular Pyrex vessel (26 cm × 17 cm, height 5 cm) under 2.5 cm of saline.

The rectangular Pyrex vessel containing the blood vessels was transferred to a 150-L hyperbaric chamber and placed on a pair of the welded aluminum plates, with cooled water circulating in the space between the two plates. Cooling during the hyperbaric exposure was necessary for the tissue preservation. The temperature of the circulating water was 6°C. Saline temperature was 1°C compared to the circulating water. Pressure was elevated (by using air) at a rate of 200 kPa/min to 1,013 kPa, 90 m sea water, and remained at that pressure overnight (∼19 h). In the following morning, the chamber was decompressed at a rate of 200 kPa/min. The rectangular vessel was placed carefully on a heating pad (E12107819, Sunbeam, Hong Kong, China) for photography. Saline was gently mixed to let the upper, gas-rich layer, mix with the deeper layers. This study began automated photographing at 5-s intervals, 15 min after the end of the decompression, for a period of 30 min (appropriate for the detection of the AHS) (Arieli and Marmur, 2016) for the AFM protocol and 60 min (allowing more time for the detachment of the bubbles) for the other two protocols. At the end of this photographic session, the rectangular vessel was kept in the refrigerator. Active hydrophobic spots can be determined by observing the formation of the bubbles after the decompression.

The photographs of each sample were examined in sequence for the appearance of the bubbles. At the end of the observation, the location of the AHS and the number of bubbles produced at each AHS were noted on the photograph that had been prepared for use during tissue sampling. Both locations of the AHS and control (area distanced from an AHS) were marked on the photograph to be sampled for the microscopy. For a control for the LM in order to exclude the effect of the detaching bubbles and prolonged hyperbaric exposure, blood vessels were prepared as previously described and under saline (without hyperbaric exposure), it was taken for the LM sampling.

## Methods and Results

### Atomic Force Microscopy Study

In this study, we aimed to observe the nanobubbles at the AHS.

In the day following decompression, the rectangular vessel with the blood vessels under saline was transferred to the AFM department in Weizmann Institute of Science, Rehovot, Israel.

Measurements were made in a JPK NanoWizard 3 AFM by using the qp-BioAC-CI probes from the Nanoworld. Custom cells were prepared to house the sample and the liquid while fitting the dimensions of the AFM. The AFM topographical measurements were attempted in the two different modes. The first mode is the AC mode, where the probe is excited at its resonance frequency and the amplitude is controlled, as it taps on the surface by a feedback, which provides the height changes, i.e., topography during the scan. The second mode is the QI mode where the probe is also oscillated, but not at the resonance frequency, but rather at a lower rate, two to three orders of the magnitude slower. In this case, not the amplitude, but the peak force applied on the sample is controlled to get the topography. The QI mode was found to have better control over the dynamics and made it possible to acquire some images. The plates moved around within the saline-containing cell, but it was possible to fix them in place by using an underwater epoxy. After this, some images could be obtained but they were of poor quality because the sample is extremely rough and has loose tissue and debris on the surface. A further attempt after storing the sample in the refrigerator for a couple of days was futile because the amount of debris in the saline solution had increased and the AFM signal (which is based on optical reflection) was not stable, even when the probe was not touching the surface.

**FIGURE 1:**
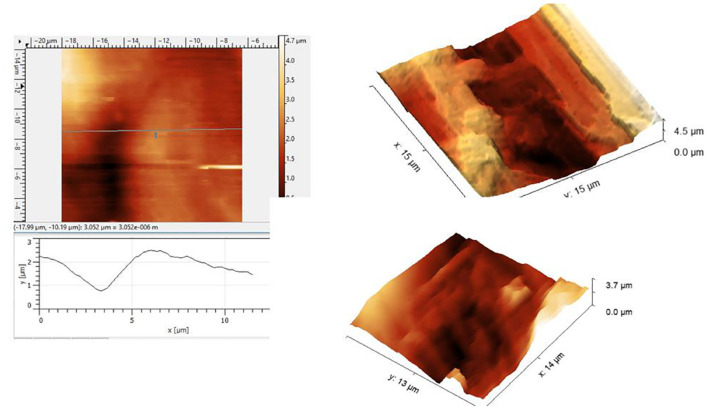
Atomic force microscopy (AFM) scans under the saline of the luminal aspect of the blood vessels at the active hydrophobic spot (AHS) sites. Rough surface disables a closer approach to the sensing probe.

A few representative images are given in Figure 1 showing the roughness of the surface. The roughness should be significantly smaller than the expected 100 nm size of the nanobubbles. The optical images are at a very large scale relative to the scanning size of the tens of microns. Nanobubbles could not be demonstrated because of the roughness and the debris at the AHS. Therefore, the observation of the control area was of no use. The failure of the AFM attempt led us to continue with the electron microscopy study to look into the roughness of the surface and possible breaches in the AHS.

### Electron Microscopy Study

The aim of the EM study was to observe the damage to the endothelial cell caused by the detaching bubble. This study expected to observe the breaches in the oligolamellar phospholipids (AHS) at the outer membrane of the endothelial cell. Blood vessels were extracted from the saline for direct sampling. Samples from the areas near the AHS and the locations distant from an AHS were dissected from the blood vessels of the sheep and were prefixed with 2.5% glutaraldehyde in 0.1 M phosphate buffer (PB) (pH 7.4) for 1 h and postfixed with 1% osmium tetroxide in 0.1 M PB for 1 h. The specimens were routinely dehydrated by passing the tissue through a series of solutions with the increasing ethanol concentrations and then embedded in the Epon 812 epoxy resin. To examine the specimens, the thick Epon sections were first cut at 0.5-μm thickness and routinely stained with toluidine blue. Then, the ultrathin sections were cut at 70–80-nm thickness with a diamond knife on an ultramicrotome and mounted on Φ1 mm × 2 mm single-slit copper carbon grids. The ultrathin sections of the tissues on the copper grids were double stained with uranyl acetate and lead citrate and, finally, observed under a JEOL 1011 transmission electron microscope (JEOL Ltd., Akishima, Tokyo, Japan) at an accelerating voltage of 80 kV.

**FIGURE 2:**
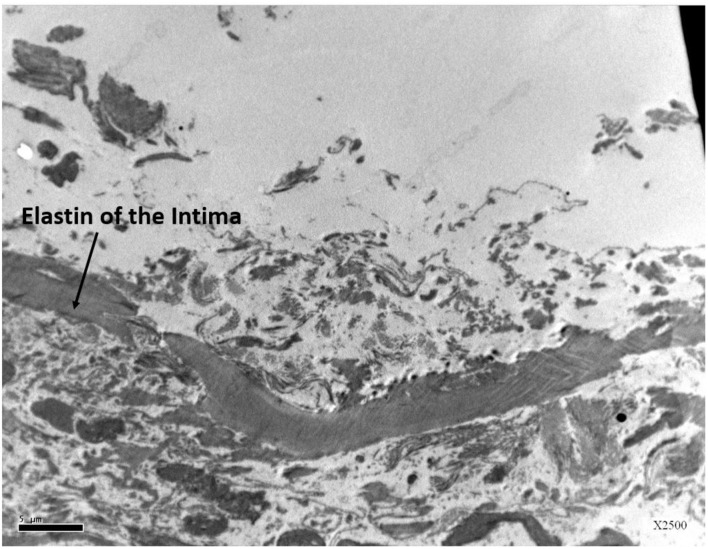
Electron microscopy view of the luminal aspect of the blood vessels at the AHS sites. Only the elastin layer remained from the intima where the endothelial cells are missing.

**FIGURE 3:**
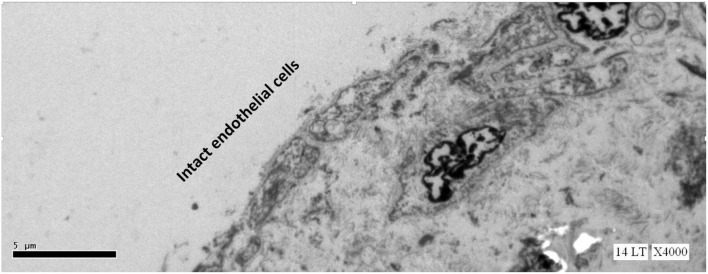
Electron microscopy view of the luminal aspect of the blood vessels at a control site (away from the AHS). Intact endothelial cells are seen.

This study expected to observe the breaches in the oligolamellar lining of the phospholipids at the AHS. However, in the first few samples from the AHS, no endothelial cells were seen at the luminal side of the blood vessel. For denial of the wrong sampling, this study let the professional staff at the pathology department to make the sampling. Again, no endothelial cells were observed, but the elastin layer of the intima adjacent to the media was observed at the AHS (Figure 2) with some debris from the teared endothelial layer. Intact endothelial cells were observed only in the samples of the control area, distant from the AHS (Figure 3). Detaching bubbles teared off the entire endothelial layer at the AHS. Detachment of an entire endothelial layer led us to turn to observation with the LM.

### Light Microscopy Study

Blood vessels were extracted from the saline for direct sampling. Samples from the areas near the AHS and sites distant from the defined AHS were dissected from the blood vessels of the sheep and the samples from the blood vessels were prepared in the same way without exposure to the hyperbaric conditions. The luminal aspect of the blood vessel, exposed to the hyperbaric chamber, was stained green for later identification. For the observation of the endothelial cells, a typical marker CD31 was stained. CD31-specific immunohistochemistry (IHC) (clone JC/70A) was performed on 3-μm thick paraffin sections of the tissue blocks by using the Ventana BenchMark XT automated slide-staining system (Ventana Medical Systems Incorporation, Tucson, Arizona, United States) as recently described: After the cell conditioning with the cell conditioner 1 (Ventana Medical Systems Incorporation, Tucson, Arizona, United States) for 32 min and the preprimary peroxidase inhibition, the slides were incubated with the commercially available antibodies (dilution 1:70; Thermo Fisher Scientific, Kalamazoo, Michigan, United States) for 32 min at 37°C. Primary antibodies were detected by using the iView DAB IHC Detection Kit (Ventana Medical Systems Incorporation, Tucson, Arizona, United States). Slides were counterstained with hematoxylin for 2 min each. Blood vessels, which were not exposed to the hyperbaric conditions, were stained with the standard H&E.

**FIGURE 4:**
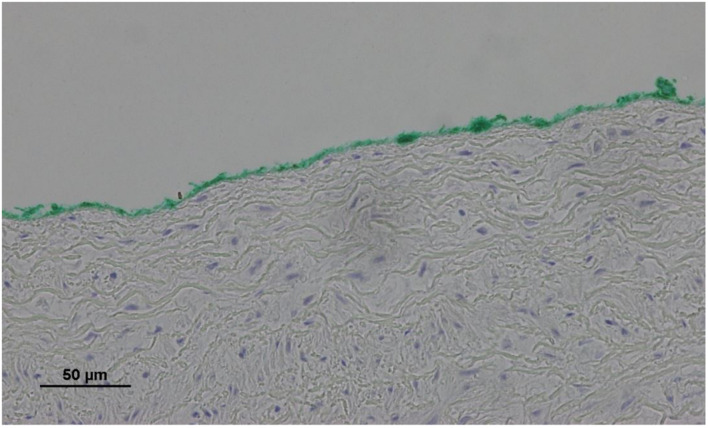
Light microscopy view of the luminal aspect of the aorta at the AHS. Green line denotes the luminal aspect. There are no endothelial cells.

**FIGURE 5:**
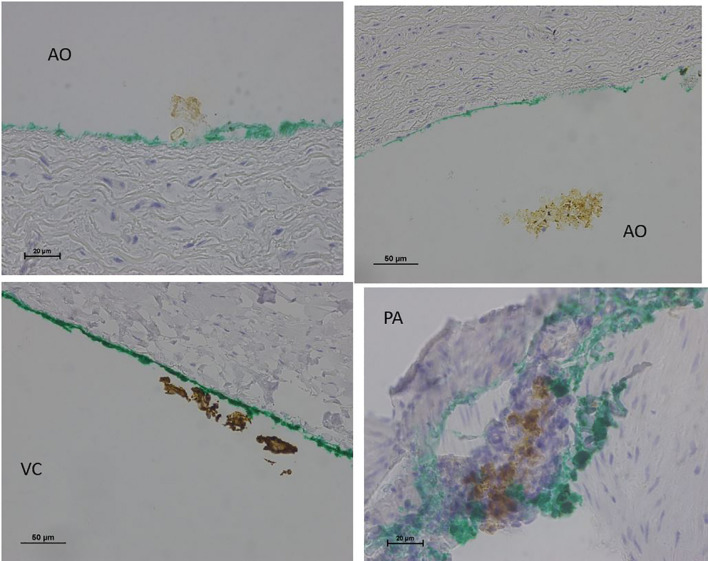
Light microscopy view of the luminal aspect of the blood vessels: aorta-AO, superior vena cava-VC, and pulmonary artery-PA at the AHS. Green line denotes the luminal aspect. There is debris or detached cluster of the endothelial cells (brown color).

**FIGURE 6:**
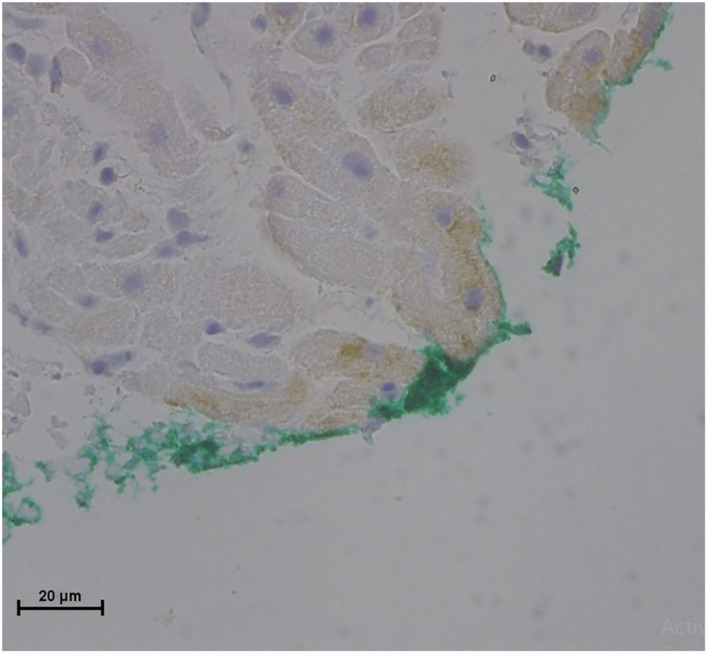
Light microscopy view of the luminal aspect of the superior vena cava away from the AHS. Green line denotes the luminal aspect. There are intact endothelial cells.

**FIGURE 7:**
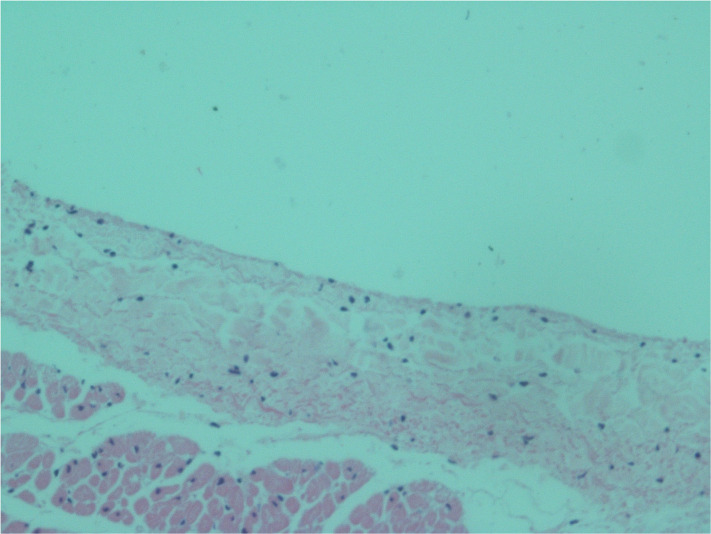
Light microscopy view of the luminal aspect of the unexposed superior vena cava. There are intact endothelial cells.

No endothelial cells were observed in the hyperbaric exposed blood vessels at the areas near the AHS (Figure 4). In some sections, debris or a detached cluster of the endothelial cells were observed (Figure 5). Intact endothelial cells could be found at the sites distant from an AHS (Figure 6). Intact endothelial cells were observed in the control unexposed blood vessels (Figure 7).

## Discussion

In this study, the main outcome is that the detached bubble at the AHS tears off the entire underlying endothelial layer. Subsequently, it causes endothelial damage. The rough surface, which remained after 75 min of the detaching bubbles, is inaccessible to the close approach of the AFM probe. Thus, our previous suggestion (Arieli, 2017) that the bubble detaches from the AHS with a section of the endothelial membrane is supported. A suggestion similar to this study was made by the Madden and Laden (2007): *Gas bubbles released into the circulation will interact with the surface of the vascular endothelium and may give rise to a measurable response linked to the state of the endothelium*. The only difference is that this study considers the local (and not circulated) bubbles at the endothelium.

Klinger et al. (2011) presented an injury in the contact of the bubbles with the umbilical endothelium. They also showed that the addition of the surfactant prevented the damage. It is well possible that the hydrophobic tail of the surfactant clings to the hydrophobic AHS and, thus, the hydrophilic head of the surfactant prevents the attachment of the bubbles and the affected injury. Sobolewski et al. (2011) found a reduction in the endothelial injury when surfactant was added before the contact of an air bubble with a single endothelial cell. Surfactant also reduced the bubble adhesion in the arterioles and the damage produced with the injected microbubbles (Suzuki et al., 2004).

Thom et al. (2018, 2019) suggested that the microparticles induce the vascular injury. However, Lambrechts et al. (2017) proved that the vascular dysfunction is related to the decompression bubbles and not to the same diving protocol almost without the decompression bubbles (predive vibrations). Microparticles may induce further injury, but the initial injury is related to the detached bubbles. Zhang et al. (2017b) presented a high correlation between the markers of the endothelial injury and the total amount of the bubbles and the time course of the bubbles in the decompressed mice. Endothelial markers and bubble score peaked at about half an hour and the markers outlasted the bubbles (<2 h) at 12–24 h. The good correlation suggests a cause-and-effect relationship and the outlasting endothelial injury points to further damage, which may be induced by the microparticles. Nossum et al. (1999) exposed the pigs to high pressure. Endothelial damage, expressed as the total response of the pulmonary artery to the substance P, was reduced only in the pigs with the recorded bubbles in the pulmonary artery, but not in the others. In their recent study, Yu et al. (2017) showed that the bubbles induced the endothelial microparticles in the cultured pulmonary endothelial cells from the rat that further caused the endothelial injury in both the cultured endothelial cell and the whole animal.

This study supports the assumption where the detached bubbles tear off the endothelial cells and cause the initial endothelial injury and agree with the conclusion of Zhang et al. (2017b): *bubbles are the most likely initial causative agents of the endothelial dysfunction following the diving decompression*.

Many of the manifestations of the DCI were related to the endothelial microparticles (Thom et al., 2011, 2012, 2013; Yang et al., 2012). We suggested that a detached venous bubble carries with it the endothelial membrane and after releasing most of its gas in the lung, it becomes a gas-containing endothelial microparticle (Arieli, 2017). This study suggests that the endothelial membranes are carried with the detached bubbles, which at least support the first step in the origin of the decompression microparticles.

## Data Availability Statement

The raw data supporting the conclusions of this article will be made available by the authors, without undue reservation.

## Author Contributions

The author confirms being the sole contributor of this work and has approved it for publication.

## Conflict of Interest

The author declares that the research was conducted in the absence of any commercial or financial relationships that could be construed as a potential conflict of interest.

## Publisher’s Note

All claims expressed in this article are solely those of the authors and do not necessarily represent those of their affiliated organizations, or those of the publisher, the editors and the reviewers. Any product that may be evaluated in this article, or claim that may be made by its manufacturer, is not guaranteed or endorsed by the publisher.
